# Commonly occurring adversities in families as risk factors for developing psychosocial and psychiatric morbidities: evidence from general practice

**DOI:** 10.1192/bjo.2022.511

**Published:** 2022-06-30

**Authors:** Indira Tendolkar, Talip Polat, Hans Peters, Reinier Akkermans, Floris van de Laar

**Affiliations:** Donders Institute for Brain, Cognition and Behavior, Centre for Medical Neuroscience, Department of Psychiatry, Radboud University Nijmegen Medical Center, The Netherlands; Department of General Psychiatry, Institute for Mental Health Care Eindhoven (GGzE), The Netherlands; Department of Primary and Community Care, Radboud University Nijmegen Medical Center, The Netherlands; Department of Primary and Community Care, Radboud University Nijmegen Medical Center, The Netherlands; Department of Primary and Community Care, Radboud University Nijmegen Medical Center, The Netherlands

**Keywords:** Childhood experience, comorbidity, outcome studies, primary care, risk assessment

## Abstract

**Background:**

Childhood adversity may lead to mental and somatic complications throughout life. General practitioners are equipped to identify and manage adverse events in households. The relationship between adversities and psychiatric symptoms has not been studied in primary care.

**Aims:**

We investigated the relationship of common adversities in families with respect to subsequent development of psychosocial and psychiatric problems in young children.

**Method:**

We analysed data from seven general practices, including participants between 0 and 9 years of age. Adversity was defined as having a household member who was diagnosed with cancer, psychiatric disease or social problems. We compared these patients with controls matched for gender, age and general practice. The primary outcome was any new episode defined with a psychological and psychiatric label. Secondarily, the encounter rates at the general practices after adversity were analysed.

**Results:**

Participants in both groups were followed for an average of 12 years, whereby patients with an adversity were more likely to develop psychiatric morbidities compared with matched references (odds ratio 1.38, 95% CI 1.12–1.68, *P* = 0.002), also revealing higher encounter rates at general practices. We found no statistically significant association between adversities in the family and increased psychosocial symptoms.

**Conclusions:**

The short- and long-term consequences of exposure to negative events in childhood are of great public health importance. Our data suggest screening more proactively for consequences of commonly occurring adversities in families, as they are a risk factor for subsequent psychiatric symptoms. Enhanced consultation frequency at general practitioners following adversities should be differentiated in more detail.

There are significant relationships between adverse life events, psychosocial resources and mental and somatic well-being throughout the lifespan. The earlier adverse events occur in life, the more likely they are to affect all subsequent levels of mental and physical development. Childhood adversity promotes subsequent health problems through pathways, including physiological challenge of the hypothalamic–pituitary–adrenal axis and social mechanisms, both of which affect the brain development (see Nelson et al^1^ for a recent review). It has been acknowledged that mental health services for children and adolescents are chronically underfunded and undervalued,^2^ despite 50% of mental illness beginning before the age of 14 years and 75% by the age of 24 years.^3^ Early identification is thus an important prerequisite for a successful prevention of long-term consequences of childhood adversity in children. Recently, Nelson and colleagues^1^ have suggested to broaden assessment of interventions beyond mental health measures, and include stress-related health outcomes such as asthma, infection, inflammation and insulin resistance. Primary care in general practices could play an important role in this,^4^ and there are several actions that general practitioners (GPs) can take to help improve outcomes.^5^

Thus far, the emphasis in monitoring in GP practices seems to have been on more severe adversity,^2^ but translational studies have shown that more subtle indirect adversities may also do great harm and are possibly not screened for often enough. Among more general psychosocial circumstances, cancer in household members has been identified to be an important risk factors (for review, see Walczak et al^6^). For example, Huizinga and colleagues^7^ have shown that parental cancer is a significant inducer of psychological fragility in terms of internalising and cognitive problems in children. Likewise, the impact of severe family distress owing to parental psychiatric disorders has been investigated, often with respect to the gene×environment interaction effect on developing the same problems later in life.^8^ Other more general psychosocial problems include, for example, stress of parents at work. Moreover, we know that limited financial resources and workplaces with fewer family-friendly policies are risk factors for family life and child development.^9^

## Aims

The overall goal of this study was to investigate the difference in occurrence of psychosocial and psychiatric problems in children who experienced indirect forms of frequently occurring early adversity, compared with matched controls. To realise this aim, we analysed data from a prospective database of GPs, allowing us to look at the course of these patients in their primary care setting. The form of registration in primary care allowed us not only to identify episodes of morbidities after adversity that were registered in primary care, but also all morbidity episodes that were registered in secondary and tertiary care and reported back to the GP. We hypothesised that children exposed to adversity were more likely to develop psychosocial and psychiatric complications and visit their GP more frequently than matched control patients from the same practices.

## Method

### Data source

This retrospective study of prospectively collected data was conducted on the basis of the Family Medicine Network (FaMe-Net database^10^) in The Netherlands. The Dutch Practice-Based Research Network (PBRN) FaMe-Net, the world's oldest PBRN, has a long history of systematically recording all morbidity presented to the family physician in episodes. FaMe-Net is a practice-based research network from the Department of Primary and Community Care at the Radboud University Medical Center, which aims to contribute to research and education and thereby improve the quality of primary care. FaMe-Net–associated physicians systematically and prospectively register data electronically on the reason for encounter, diagnostic procedures, diagnoses, interventions and referrals. All data in this database are anonymised and patients in FaMe-Net gave permission to use data for research purposes by an opting-out procedure. All doctors are trained in registration as part of belonging to the network. There is regular supervision and quality control on the registration of the data. Diagnosis in all patient encounters are coded by the GP, according to the International Classification of Primary Care (ICPC-2^11^), extended by the ICD-10.^12^ In this network, which consists of seven Dutch family practices in the East Netherlands, all encounters between family practices and patients are registered since 1971. All information belonging to one health problem is ordered in one episode, whereby information from other institutions and specialised diagnostics are included to support diagnoses.

### Extraction rules

In this study, patients were included who encountered an indirect adversity before the age of 9 years. To this end, data were extracted from 1 January 1995 until 31 December 2016, whereby a potential adversity occurred before 2013, such that minimum age of inclusion was 4 years. We defined an indirect adversity as the patient having a household member diagnosed with one of the following conditions: cancer, a severe psychiatric diagnosis or social problems (indexed in Table 1). Because of the given evidence in the literature, we defined a hierarchy whereby cancer was taken as the primary adversity, followed by psychiatry diagnosis and then social problems, which are more frequently reported in GP practices and can be the least specific in a database. To account for potential mistakes owing to double registrations, we selected one household member (i.e. family member) with an indirect adversity for each patient. The follow-up period was determined as a minimum of 4 years since the first experience of adversity. An event for a family member subsequently leading to indirect adversity in our patient group could therefore potentially have occurred in the recent period before the birth of that patient, so that the adversity ‘started’ at the age of 0 days.

**Table 1:** Overview of the classification of the indirect adversities, according to the International Classification of Primary Care, Second Edition (ICPC-2)

Code	Definition
1. Cancer in household members	
A79	Malignancy not otherwise specified
B72	Hodgkin's disease/lymphoma
B73	Leukaemia
B74	Malignant neoplasm blood other
D74	Malignant neoplasm stomach
D75	Malignant neoplasm colon/rectum
D76	Malignant neoplasm pancreas
D77	Malignant neoplasm digest other/not otherwise specified
F74	Neoplasm of eye/adnexa
H75	Neoplasm of ear
K72	Neoplasm cardiovascular
L71	Malignant neoplasm musculoskeletal
N74	Malignant neoplasm nervous system
N76	Neoplasm nervous system unspecified
R84	Malignant neoplasm bronchus/lung
R85	Malignant neoplasm respiratory, other
S77	Malignant neoplasm of skin
T71	Malignant neoplasm thyroid
U75	Malignant neoplasm of kidney
U76	Malignant neoplasm of bladder
U77	Malignant neoplasm urinary tract
X75	Malignant neoplasm cervix
X76	Malignant neoplasm breast female
X77	Malignant neoplasm genital other
Y77	Malignant neoplasm prostate
Y78	Malignant neoplasm male genital other
W72	Malignant neoplasm relate to pregnancy
2. Psychiatric diagnoses of household members	
P71	Organic psychosis other
P72	Schizophrenia
P73	Affective psychosis
P74	Anxiety disorder/anxiety state
P77	Suicide/suicide attempt
P80	Personality disorder
P82	Post-traumatic stress disorder
P86	Psychological disorders, other
3. Social problems of household members	
A96	Death
Z01	Poverty/financial problem
Z02	Food/water problem
Z03	Housing/neighbourhood problem
Z04	Social cultural problem
Z05	Work problem
Z06	Unemployment problem
Z07	Education problem
Z08	Social welfare problem
Z09	Legal problem
Z10	Healthcare system problem
Z11	Compliance/being ill problem
Z12	Relationship problem with partner
Z13	Partner's behaviour problem
Z14	Partner illness problem
Z15	Loss/death of partner problem
Z16	Relationship problem with child
Z18	Illness problem with child
Z19	Loss/death of child problem
Z20	Relationship problem parent/family
Z21	Behaviour problem parent/family
Z22	Illness problem parent/family
Z23	Loss/death parent/family member
Z24	Relationship problem friend
Z25	Assault/harmful event problem
Z27	Fear of a social problem
Z29	Social problem not otherwise specified

The control group were matched to the index group for gender, age and the general practices. Matched references were not diagnosed with cancer or a social problem, and did not have a family member that was diagnosed with cancer, a psychiatric disease or a social problem. Importantly, also with respect to the time frame of the observed results, the matched reference was selected based on the date of indirect adversity diagnosis of the index patient, to analyse potential psychosocial and psychiatric problems in the same time period. We included index patients with a minimum of one and maximum of four matched control patients, to have more data to calculate statistics.

Extracted data for this study were age, gender, practice, diagnosis coded by ICPC codes, date of diagnosis, date of practice registration or deregistration, and the reason for deregistration.

The Radboud University Medical Center's Technology Center Health Data supports FaMe-Net in distillation and secure storage of routine data from the affiliated general practices. It adheres to the regulations of Dutch and European laws, and has gained ethical approval from the research ethics committee of Radboud University Medical Center for this procedure (medical ethics number 2020-6871).

### Analyses

The primary outcome variables were new ICPC-2 ‘P-episodes’ (for an overview, see https://ehelse.no/kodeverk/icpc-2e–english-version).^11^ We distinguished episodes with psychiatric or psychosocial symptoms only (ICPC codes P1–P29), and episodes resulting in a psychiatric diagnosis (ICPC codes P70–P99), as can be found in Table 2. We selected the first psychiatric and psychosocial complications that were reported in the follow-up period after the adversity, whereby we adhered to the hierarchy that subsequent report of psychiatric diagnoses was noted before psychosocial symptoms, as this was thought to reflect the more clinically relevant outcome.

**Table 2:** Overview of the odds ratio of development of psychiatric diagnosis and psychosocial symptoms in the group that encountered indirect adversity and the control group

	Patients (*n* = 1029)	Controls (*n* = 3849)	Odds ratio	*P*-value	95% CI (mid *P* exact)
Number of patients receiving at least one:					
Psychiatric diagnosis	156 (15.2)	465 (12.1)	1.38^a^	0.002	1.12−1.68
Psychosocial symptoms	304 (29.5)	1117 (29.0)	1.04	0.620	0.89−1.21

a. Significant confidence interval.

The secondary outcome variable was the number of visits at the GP practice in a follow-up period of at least 4 years since the first adversity event. Consultations for all different ICPC codes were counted for this rate. We also calculated the different follow-up length in terms of years after the index event.

Data management and analysis were performed with SPSS version 22.0 (SPSS Inc., Chicago, Illinois, USA) software for Windows. Descriptive statistics were used to describe the main findings. Given that our primary outcome was associated with a dependent variable with two possible values (yes/no psychiatric diagnosis), we opted for a binary logistic regression model to compare our group of index patients with control patients. Age and gender were taken as confounding variables and were corrected for. Negative binomial regression analysis was used to test the difference in number of encounter between index patients and control patients. A *P*-value of <0.05 was considered to be statistically significant, based on two-sided tests.

## Results

The whole database consisted of 28 659 patients from all seven practices. Based on the aforementioned selection criteria, we included 1029 patients and 3849 controls. Participants encountering adversity were followed around 12 years in the same GP practice, as shown in Table 3. There were slightly more males than females in the sample, with a median age of experienced adversity of around 4 years (Table 3). Note, however, an indirect adversity could also occur before the birth of the index patient, which was the case in 70 out of 1029 patients, in which severe social problems were the most common (see Table 3).

**Table 3:** Overview of patients encountering indirect adversity (*n* = 1029) out of the whole cohort (*n* = 28 659)

Gender	Number (%)
Male	528 (51.3)
Female	501 (48.7)
	Median age at adversity (s.d.)
All	3.44 (5)
Male	3.53 (5)
Female	3.34 (5)
Categories of adversity	Number (%)
Cancer	183 (17.8)
Psychiatry	114 (11.1)
Social problems	732 (71.1)
Gender of residential connection members that induce indirect adversity	Number (%)
All	1029
Male	528 (51.3)
Cancer	95 (18.0)
Psychiatry	66 (12.5)
Social problems	367 (69.5)
Female	501 (48.7)
Cancer	88 (17.6)
Psychiatry	48 (9.6)
Social problems	365 (72.9)
Distribution age frequency at adversity	Number (%)
Before birth	70 (6.8)
0–3 years	489 (47.5)
4–9 years	470 (45.7)
Mean follow-up in general practice since first adversity event in years	Number (s.d.)
Male	12 (9.5)
Female	12 (8.9)

Results from our primary question can be found in Table 2. Patients with indirect adversity are significantly more likely to develop psychiatric symptoms compared with controls (odds ratio 1.38, 95% CI 1.12–1.68: *P* = 0.002). We found no statistically significant association between indirect adversities and the onset of psychosocial symptoms (odds ratio 1.04, 95% CI 0.89–1.21; *P* = 0.620). Moreover, patients with indirect adversities demonstrated significantly higher encounter rates (hazard ratio 1.19, 95% CI 1.10–1.28; *P* ≤ 0.001) compared with matched controls (mean encounter per year 16.27 (s.d. 15.53) *v*. 13.94 (s.d. 13.13).

When looking at the different psychosocial and psychiatric problems (Table 4) in both groups, we found that having behavioural problems, specific intellectual disabilities and feeling anxious were the most common problems in this follow-up period. This was mirrored by the psychiatric morbidities, whereby attention-deficit hyperactivity disorder and anxiety were most common.

**Table 4:** Absolute frequencies and percentage of psychosocial symptoms and psychiatric morbidities according to the International Classification of Primary Care in patients that experienced indirect adversities and their matched controls

	Indirect adversity group (*n* = 1029)	Control group (*n* = 3849)^a^
Any symptom or morbidity	542 (52.7)	1582 (41.0)
Psychosocial symptoms		
P01 Feeling anxious/nervous/tense	43 (4.2)	155 (4.0)
P02 Acute stress reaction	8 (0.8)	38 (1.0)
P03 Feeling depressed	18 (1.7)	47 (1.2)
P04 Feeling/behaving irritable/angry	12 (1.2)	23 (0.6)
P08 Sexual fulfilment reduced	1 (0.1)	4 (0.1)
P09 Sexual preference concern	1 (0.1)	1 (0.03)
P11 Eating problem with child	7 (0.7)	27 (0.7)
P22 Child behaviour symptom/complaint	108 (10.5)	292 (7.6)
P23 Adolescent behaviour symptom/complaint	5 (0.5)	33 (0.9)
P24 Specific learning problem	129 (12.5)	444 (11.5)
P25 Phase of life problem adult	3 (0.3)	9 (0.2)
P29 Psychological symptom other	23 (2.2)	44 (1.1)
Psychiatric morbidities		
P15 Chronic alcohol abuse	0	3 (0.08)
P18 Medication abuse	2 (0.2)	2 (0.05)
P19 Drug abuse	7 (0.7)	19 (0.5)
P72 Schizophrenia	0	0
P73 Affective psychosis	0	2 (0.05)
P74 Anxiety disorder/anxiety state	26 (2.5)	74 (1.9)
P75 Somatisation disorder	3 (0.3)	5 (0.1)
P76 Depressive disorder	19 (1.8)	56 (1.5)
P77 Suicide/suicide attempt	0	1 (0.03)
P78 Neurasthenia/surmenage	5 (0.5)	14 (0.4)
P79 Phobia/compulsive disorder	5 (0.5)	18 (0.5)
P80 Personality disorder	3 (0.3)	9 (0.2)
P81 Hyperkinetic disorder (attention-deficit hyperactivity disorder)	81 (7.9)	175 (4.5)
P82 Post-traumatic stress disorder	2 (0.2)	7 (0.2)
P86 Anorexia nervosa/bulimia	0	9 (0.2)
P98 Psychosis not otherwise specified/other	3 (0.3)	3 (0.08)
P99 Psychological disorders, other	28 (2.7)	68 (1.8)

a. For the control group, only the first (symptom) diagnosis was extracted for this study.

## Discussion

This is, to the best of our knowledge, the first study in a primary care setting that has prospectively investigated the effect of commonly occurring indirect adversities before 9 years of age on the subsequent development of psychosocial problems and psychiatric morbidity. Using a large sample of index samples and matched controls, we were able to support our hypothesis that commonly occurring adversities are related to mental stability in young children. More precisely, we found that children with a parent with cancer, a psychiatric disease or a social problem were more likely to develop psychiatric morbidity, compared with controls from the similar primary care setting and thereby likely to share the same socioeconomic setting. Hyperkinetic disorders (P81), followed by anxiety disorders (P74) and then depressive disorder (P76) were most commonly registered in the follow-up course for both groups, mimicking the prevalence rate of so-called internalising disorders at this time in the life course. Systematic reviews suggest that the reported range in the community prevalence of attention-deficit hyperactivity disorder (2.2–7.2%) reflects variation in study methodology, and the prevalence rate in our control group is in line with this prevalence.^13^ Bias in the registration of disorders cannot be entirely ruled out, but we do not see a higher prevalence of developmental disorders in the control group. When comparing the distribution in the indirect adversity and control groups, indirect adversity was not related to another distribution of psychosocial and psychiatric complications in childhood and adolescence, but was related to a higher frequency. Finally, mean encounter rates per year at primary care centres were significant higher in participants that had experienced adversities, compared with controls. Unfortunately, we were not able to differentiate whether adversity led to greater need for care in the context of psychiatric complications or somatic problems, which are also a well-documented consequence. We cannot rule out that as a result of more encounters, there was a higher likelihood of detecting problems within primary care in the indirect adversity group. However, the method of registration in the GP practices allowed us not only to identify post-adversity morbidity episodes that were registered in primary care, but also all morbidity episodes that were registered in secondary and tertiary care and reported back to the GP, so that it seems likely that these are valid diagnoses.

The onset of mental disorders in those that have experienced childhood adversity has been extensively studied with retrospective questionnaires that cover a wide array of adversities. Childhood adversity has been linked to the onset of different dimensions of stress-related psychopathology, such as depression, schizophrenia, severity of bipolar disorder and increased risk of psychosis.^14–16^ Focusing on more frequently occurring adversities, we can support this notion from the perspective of a GP, at least in the observed period of approximately 12 years after a reported episode. Although there were limitations in assessing all kinds of chronic stress in the families, our findings support other studies showing that children with parental cancer in the age group of 11–23 years have reported internalising and cognitive problems.^7^ It has been acknowledged that GPs should be alert for somatic and psychosocial problems in partners of patients with cancer, but studies prospectively looking into the effects on children are sparse. Thastum and colleagues^17^ investigated a cohort of children and adolescents whose parents were suffering from cancer. The authors showed a higher risk of problems, particularly when the father was ill, but it remains unclear whether this difference was because of the different diagnoses of fathers and mothers, gender or other factors. Moreover, internalising problems in children and adolescents were best predicted by parental depression, whereas family dysfunction was related to externalising problems in the offspring. Chen and Parebianco^18^ showed that emotional well-being of ill parents was directly associated with adolescent distress, which also led to mental health problems later in development.

In the present study, we explicitly tried to include the concept of indirect adversity because longer-lasting stressful events in the household are an important risk factor even when children are not directly affected. Our study is limited by the fact that the relationship between the affected child and household members is not well defined. Yet, we know that stress impact also occurs as any household member may induce psychological and health problems. Of course, we have to acknowledge that the concept of an indirect adversity is difficult to define in a naturalistic cohort setting. We cannot clearly identify the length and severity of the indirect adversity period. Indirect adversity, in our case, could even occur before birth, and thus longer-lasting programming effects on the developing brain may have effects ranging from stunted physical growth and cognitive delays to problems regulating attention.^19^ As is also evident from the demographics, most of the patients we investigated were still in their early adolescence during the time of the follow-up in this study. Based on the epidemiological evidence, it is likely that more mental health problems occur later on in life, so we can only reflect on the impact at that point. From naturalistic cohorts in adulthood, we know that the frequency of childhood adversity of any kind was positively associated with psychiatric comorbidity.^20^

Currently, interventions informed by specific trauma in early life have not yet been adapted from mental health settings for use in primary care. A few studies have investigated the use of additional screening lists in primary care. Notably, however, such questionnaires mainly covered more severe forms of adversity and not the common psychosocial episodes we have investigated. For example, Glowa and colleagues^21^ used a ten-item childhood adversity questionnaire as a screening tool in a family practice setting, with patients presenting for follow-up of chronic illness or annual physicals. Based on the sum score, they divided patients with higher and lower risk scores and found that based on the outcome scores, clinicians were more likely to have discussed adversity issues for high-risk patients. If these data could be replicated in larger samples, they could lead to policy changes in staging risks after such events, promoting more preventive actions in primary care. This is of relevance because in many countries, secondary mental healthcare comes with long waiting lists and area-specific availability. As there is an ongoing concern that longer waiting times for treatment leads to poorer health outcomes, earlier interventions in primary care, if possible, could circumvent side-effects of restricted specialised care. In fact, all of the psychiatric morbidities we found in the follow-up period of the children with indirect adversity show high comorbidity with somatic pathology, such as cardiovascular disease, rheumatoid arthritis and diabetes.^22,23^ Such comorbidity is associated with unfavourable outcomes for the individual, such as low quality of life^24^ and mortality.^25^ Not surprisingly, it leads to high levels of healthcare utilisation,^26^ which was also supported by increased encounter rates with the GP practices in our data-set.

### Strengths and limitations

A strength of this study is the large size of our study population and the longitudinal primary care data investigating the effect of childhood adversity on psychiatric morbidities and psychosocial symptoms, using ICPC-2 codes. Many studies have investigated the effect of adversity concerning only one psychiatric diagnosis, e.g. depression, schizophrenia, bipolar disorder and psychosis. Lastly, the follow-up period of this study has an average of 12 years, which is long enough to provide clear information and draw conclusions about our findings.

This study also has a number of limitations. We included residential connection members (e.g. investigating parental cancer), assuming that they are family members. However, the precise relationship of a patient with their household member is not registered in our database. Yet, we also know that stress effects can occur more broadly within a whole household/family, and in this pilot analysis, we particularly focused on more severe and disabling psychiatric diagnoses of household members. Depression and substance use disorders are also highly prevalent^27,28^ and can be disabling, but in the ICPC system, no distinction is possible between (frequently occurring) mild depression and the level of alcohol (mis)use. Future studies will need to disentangle risk for indirect adversity in more detail. To assess potential causality in more depth, these studies should prospectively collect more detailed data on family and socioeconomic circumstances, as well as the impact of indirect adversity (e.g. severity of the event). It is known that all patients lived with the person experiencing adversity. Moreover, a household member in the context of the registration in the GP system is, per definition, meaningful because they live together and the definition of a household member within the GP system excluded incidental circumstances where, for example, somebody else is temporarily living as a lodger at the same address. Another limitation is that the encounter rates for different adversity groups could be overestimated, since the registration date of an ICPC-2 code is used as the start date of an adversity. Selecting only one household member (i.e. family member) with indirect adversity for each patient did not allow us to dissociate the quantitative effects in further detail, such that other family members could have also experienced an event that may have led to indirect adversity. Further, the comparison between absolute frequencies of (symptoms) diagnoses between the two groups was hampered by the fact that for the control group, only the first diagnosis was extracted. However, this did not affect the primary outcome because this was based on the occurrence of any first outcome. Finally, a limitation can be the possibility of incomplete data entry, as in any large administrative data-set; however, the necessity for GPs to indicate the episode correctly with regards to qualitative checks and financial controlling helps to minimise this risk.

In sum, our data show that common indirect adversities a child can experience in their household is an important risk factor for developing psychosocial and psychiatric morbidities in later childhood and adolescence. Further research also using prediction models of these more common indirect adversity types would be of value to determine the high-risk population that would benefit from direct intervention in primary and potentially mental healthcare. In particular, our results may be of relevance in countries with a strong primary care system, such as the UK and The Netherlands. The findings support a systemic approach for both adults who present with cancer, severe psychiatric disorders and social problems, and for the children who may present to a higher extent with associated psychiatric problems. Moreover, psychiatric expertise might be helpful in caring for families who experience adversity, thus making a case for interdisciplinary care.

## Data availability

The data that support the findings of this study are available from the corresponding author, I.T., upon reasonable request. The data are not publicly available as they contain information that could compromise the privacy of research participants.
